# Herbal medicine used by the community of Koneba district in Afar Regional State, Northeastern Ethiopia

**DOI:** 10.4314/ahs.v21i1.51

**Published:** 2021-03

**Authors:** Ali Zeynu, Tigist Wondimu, Sebsebe Demissew

**Affiliations:** 1 Semera University, Biology Department, Afar, Ethiopia. alizeynu@gmail.com; 2 Addis Ababa University, College of Natural and Computational Sciences, Department of Plant Biology and Biodiversity Management; P.O. Box 30251, Addis Ababa, Ethiopia. tigist.wondimu@aau.edu.et/twtigistw@gmail.com; 3 Addis Ababa University, College of Natural and Computational Sciences, Department of Plant Biology and Biodiversity Management; P.O. Box 3434, National Herbarium of Ethiopia, Addis Ababa Ethiopia. sebsebe.demissew@aau.edu.et/ sebseb.demissew@gmail.com

**Keywords:** Ethnomedicine, informant consensus, snakebite

## Abstract

**Background:**

Pastoral communities of the Afar people in northeastern Ethiopia use medicinal plants for various health problems. However, very limited scientific documents are found addressing ethnomedicinal knowledge of the community.

**Objective:**

This study aimed at documenting herbal medicine and the associated knowledge from Koneba district of Afar Regional State, Ethiopia.

**Methods:**

Purposive sampling method was used to select study sites and key informants. General informants were selected through simple random sampling methods. Semi-structured interviews and guided field walk were used to collect data while Informant Consensus Factor (ICF), Fidelity Level (FL) and Preference Ranking were used to analyze and verify data.

**Results:**

A total of 67 medicinal plant species used to treat humans and livestock ailments were recorded and collected. Thirteen medicinal plant species were mentioned as effective medicine against snake bite (ICF; 0.68) while nine species used to treat malaria, common cold and fever (ICF: 0.67). *Cyphostemma adenocaule* (Steud. ex A.Rich.) Desc. ex Wild & R.B.Drumm. was the most preferred species used to combat snakebite, which was prevalent in the area.

**Conclusion:**

Snake bite, malaria, common cold and fever are common health problems in the study area. Efficient use of herbal medicine has minimized the impact of these diseases.

## Introduction

The practice of herbal medicine has a long term history and culture among many African communities. This persistent interaction of people and herbal medicine is mainly due to recognition of healing effects of the system. 1, 2 Knowledge and use of medicinal plants in Ethiopia play important role in the primary health care needs for both human and livestock. A number of ethnomedicinal studies documented this vital knowledge representing different communities from northern, southern and central parts of the country. For example:^3^, ^4^, ^5^, ^6^, ^7^, ^8^, ^9^, ^10^, ^11^, ^12^, ^13^, ^14^, ^15^. Relatively, few studies have been conducted in the northeastern 16, 17, 18 and eastern 19, 20 parts of Ethiopia. These are areas where most of the pastoral community of Ethiopia reside.

The Afar Regional State lies in the arid and semiarid climatic zone of the northeastern Ethiopia within the Great Rift Valley. The topography varies from hilly escarpment in the western edge to lowland plain areas in the eastern part. According to 21, the vegetation type is predominantly Acacia-Commiphora woodland and bushland in western part, small forest patches covered with Juniperous-Olea forest in northwestern part, and desert and semi-desert vegetation type in eastern plain land.

The Afar people are among the cultural and pastoral communities in Ethiopia who have developed knowledge on herbal medicine through long time interaction with nature. Their life style coupled with different life challenges, such as limited access to modern health care^22^ has necessitated extensive use of plant resources as medicine. Despite this, very limited scientific studies have been conducted to retrieve the ethnomedicinal knowledge of the community. To-date, only 16, 17 and 18 are the ethnomedicinal studies known from the Afar Region. Such scientific evidences help to enhance contribution of nature to development of herbal medicine. 23 However, the current trend of biodiversity loss may affect availability of herbal species and the associated knowledge. This may also be accelerated by alteration of life style. 24 Therefore, retrieval and documentation of such knowledge systems before they disappear is important.

The current study was conducted in Koneba district of the Afar Regional State with the aim of documenting medicinal plant species and the associated knowledge. The study also anticipated to see the role of protected areas and home gardens in the conservation of medicinal plants and securing the knowledge system.

## Methods

### Study area

**Figure 1:**
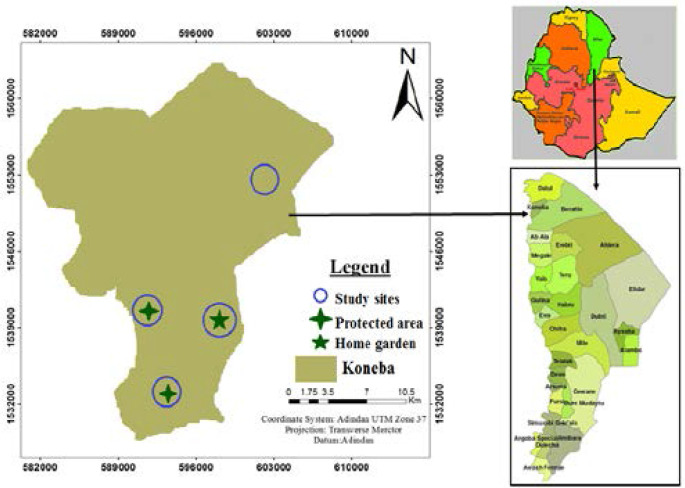
Study area and sampling sites

The Afar Regional State is located in geographic location between 8°49′ and 14030′ N latitude and 39°34′ to 42°28′ E longitude, northeastern Ethiopia. Koneba district is one of the 28 administrative districts, which is located in the northwestern part of the Regional State. It is about 624 km away from Addis Ababa, bordered by Tigray Regional State from the west, by Dallol district from north, and by Berhale district from east (Figure 1). The average elevation in this district is 1150 meters above sea level. 25

The population of Koneba is about 54,198 people who occupy, an area of 483.16 km^2^. 26 The majority of the local communities depend on livestock products and small-scale irrigation farming, which involves production of cereals, fruits and vegetables. A small proportion (0.68 %) engage in pastoral and urban (5.59 %) life styles. 26

### Sampling, data collection and analyses

Four kebeles (Smallest administrative unit in Ethiopia) were selected as study sites based on prior information from the administrative offices and based on site seen during the reconnaissance survey. Vegetation cover, proximity to plant resources and herbal medicine utilization were basis of site selection. A total of 60 informants (50 males and 10 females) between the ages of 25 and 83 were selected to engage in this study. Eighteen traditional health practitioners were identified as key informants through purposive sampling technique following. 27 General informant (42) were selected through simple random method following the method by 28.

Ethnobotanical data were collected following techniques suggested in standard manuals, guidelines and protocols. 29, 30, 31, 32, 33 Formal and informal interviews were conducted individually and in groups. Active participant observation was employed in order to get firsthand information. 34 Voucher specimens of the plants were collected and deposited at the National Herbarium of Ethiopia (ETH) as well as at the Herbarium of Traditional and Modern Medicine Directorate, Ethiopian Public Health Institute (EPHI).

Descriptive statistical tools were employed to analyze and summarize the data. Informant Consensus Factor (ICF) was calculated for each medicinal plant in order to validate responses of informants. 36 The difference between number of use citation (nur) and number of species used (nt) divided by the number of use citation minus one gives the value of ICF.

$$\text{ICF}=\frac{n_{ur}-n_{t}}{n_{ur}-1}$$

These values are presented between 0 and 1, and validity of the information obtained from informants increase as the ICF value approaches to 1. Fidelity level (FL) was calculated to verify the most important medicinal plant species in the district. The percentage of informants claiming a plant species for the same purpose provides the FL% and was calculated using the following formula.

$$\text{FL\%}=\frac{\text{Np}}{\text{N}}\times 100$$

Where Np stands for the number of informants claiming a certain plant species to cure a particular disease while N is the number of informants that use the species as medicine to treat any given disease. 30

Preference ranking was conducted following 29, to identify the most preferred medicinal plant species used to treat the prevalent diseases in the area. Six medicinal plants mentioned by more than 50% of the total informants were used in this analysis. Each key informant was asked to assign the highest value (6) for the most preferred species and the lowest value (1) for the least preferred plant, and all the rest ranging between 6 and 1. The value of each species was summed up and ranked based on the total score.

## Results

### Medicinal plant diversity in Koneba district

**Figure 2:**
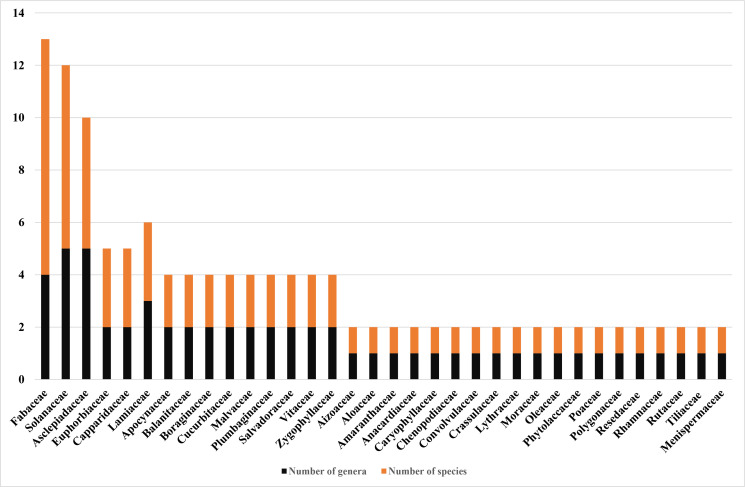
Taxonomic diversity and hierarchical composition of medicinal plants

A total of 67 medicinal plant species belonging to 58 genera and 34 families were reported in this study. The families with largest number of species were Fabaceae (9 species), followed by Solanaceae (7 species) and Asclepidiaceae (5 species). Single species per single genus was recorded in the remaining 31 Families (Figure 2). Fifty three (79.10 %) species were used to combat human ailments while 10 (15.15 %) species used to treat both human and livestock ailments. Four (6.06 %) species were reported as medicines of livestock.

### Distribution, Habit and Parts used

Thirty-three (50 %) of the species were collected from wild habitat while home gardens contributed 24 (36.36 %) species and 10 (15.15 %) species were obtained from protected areas. Analysis of diversity by habits showed 37.31 % accounting for shrubs, 35.82% for herbs and 21% for trees. The remaining 5.97% of the species were Lianas.

Leaves and roots were the most commonly used parts accounting for 91 (39.40%) and 63 (27.28%) of the remedies mentioned by the informants, respectively. Most remedies were prepared from single plant part while fourteen remedies were prepared from combinations of either leaf and root (11), leaf and stem (two), or leaf and bark (one).

### Mode of Preparation and ways of administration

Only nine percent of the mentioned mode of preparation employed dry materials. Pounding/crushing and mixing with either water or other additives were principal methods scoring 133 mentions; 57.08 % (Table 1). Water is the popular solvent used to prepare the herbal remedies (109 mentions). Honey, milk, butter, oil, animal blood, or saliva were reported as important additives (47 mentions). Oral administration accounted for the highest proportion of remedy application (114; 71.7 %) followed by topical administration (25; 15.72%). Uses of fresh materials are the most frequently mentioned methods (74.8%) while alternative use of fresh or dried materials were accounted for 17 % of preparation modes.

**Table 1:** Mode of herbal medicine preparation

Mode of Preparation	Number of mentions	Percentage
Pounding/crushing & mixing	133	57.08
Pounding/crushing	63	27.04
Boiling	11	4.72
Squeezing	10	4.29
Cooking	8	3.44
Burning	2	0.86
Others	6	2.58

### Cultural importance of herbal medicine among Koneba communities in Afar

The highest ICF (0.68) was obtained for the use of herbal medicine (13 medicinal plant species) to handle health issues related with poison, mainly with snakebite (Table 2). Nine species were mentioned to treat malaria, common cold, cough and fever scoring the second highest ICF value (0.67). The third highest ICF value (0.64) was recorded for problems related with reproductive health.

**Table 2:** Consensus factor support for disease categories reported by informants

Category	Species	Use citation	ICF
Snake bite	13	39	0.68
Malaria, common cold, cough, fever	9	25	0.67
Impotency in men, sterility in women, abortion	6	15	0.64
Evil eye, devil spirit, mental disorder	12	29	0.61
Arthritis, acne-vulgaris, leprosy, cellulites	15	36	0.60
Dandruff, thorn removal, weapon removal	6	13	0.58
Gastritis and stomach upsets, typhoid	15	29	0.55
Kidney infection, urine retention, hepatitis	10	20	0.53
Wound, ulcers	14	25	0.46
Fire burn, skin cut, blood clotting	5	9	0.44
Ear and eye infection, animal eye infection	9	14	0.38
Tonsil, splenomegaly	6	8	0.29
Anthrax	4	5	0.25
Cancerous diseases	5	6	0.20
Tuberculosis (TB) and related diseases	8	9	0.13

Highest FL were obtained for *Ziziphus spina-christi* (L.) Desf., *Cocculus pendulus* (J.R.Forst. & G.Forst.) Diels, *C. adenocaule* and *Balanites rotundifolia* (Tiegh.) Blatt. Good percentages of FL were recorded for *Balanites aegyotiaca* (L.) Delile, *Chenopodium album* L. and *Acalypha fruticose* Forssk. (See Table 3).

**Table 3:** Application of the most commonly used medicinal plants and their fidelity level

Plant name	Ailment category	Np	N	FL
*Z. spina-christi*	Dandruff	3	3	100%
*C. pendulus*	Fire burn	2	2	100%
*C. adenocaule*	Snake bite	2	2	100%
*B. rotundifolia*	Malaria	9	9	100%
*Balanites aegyptiaca* (L.) Delile	Wounds	7	10	70%
*Chenopodium album* L.	Urine retention	2	3	67%
*Acalypha fruticosa* Forssk.	Stomach swelling	2	3	67%
*Acacia oerfota* (Forssk.) Schweinf.	Common cold	3	6	50%
*Ehretia obtusifolia* Hochst. ex A.DC.	Liver diseases	2	4	50%
*Cucumis dipsaceus* Ehrenb. ex Spach	Arthritis	2	4	50%
*Acacia mellifera* (M.Vahl) Benth.	Eye infection	2	4	50%
*Achyranthes aspera* L.	Arthritis	2	6	33%

Among the six most cited medicinal plant species that were used against snakebite, *Cyphostemma adenocaule* was preferred most while *Zaleya pentandra* (L.) C.Jeffrey ranked last (Table 4).

**Table 4:** Preference ranking of medicinal plants used against snakebite

Medicinal Plants	Informants							Average score	Rank
	I1	I2	I3	I4	I5	I6	I7		
*C. adenocaule*	6	3	6	5	4	6	6	36	1^st^
*C. quadrangularis*	5	5	1	6	6	4	4	31	2^nd^
*Citrus aurantifolia* (Christm.)	4	6	4	3	3	5	3	28	3^rd^
Swingle									
*Nicotiana glauca* Graham	2	1	3	4	5	3	5	23	4^th^
*Commicarpus*	3	4	2	2	1	2	2	16	5^th^
*plumbagineus* A.Rich.									
*Z. pentandra*	1	2	5	1	2	1	1	13	6^th^

## Discussion

In this study, a substantial number of medicinal plant species were recorded and collected that justified the use of medicinal plants by the Afar pastoral community. The largest number of medicinal plant species collected in this study belong to the family Fabaceae. This family is the third largest angiosperm family and widely distributed globally (Encyclopedia Britannica) while it is the second largest in the Flora region.^36^ It includes many species that are economically important. 37 The families Solanaceae and Asclepidiaceae also include several species that are typical of arid environments such that of the Afar region.

The proportion of shrubs was high in the current study, which is in agreement with preveious studies.^1^, ^16^, ^4^, ^17^, ^18^ This is because of the influence from the dominant vegetation type, which is mainly Acacia-Commiphora woodland and bushland. 21 Composition of herbs was also comparable to that of shrubs, which could be the contribution of the home garden. Trees also contributed a good number of medicinal plant species, which could be the effect of protection.

The highest proportions of herbal medicines are prepared from fresh leaves and roots. This is consistent with findings of other studies. 3, 1, 7, 18 Preparing herbal medicines from fresh materials reduces the risk of losing active bioactive ingredients due to drying and poor storage. Leaves are the most usable parts in many cases of herbal medicine. This is attributed to higher concentration of bioactive ingredients produced and stored in leaves than other parts. 38, 39 Roots accounted for eleven kinds of remedies, which demonstrates the existence of bioactive ingredients in roots. 40

Honey, sugar and salt were used to provide the prepared herbal medicines taste as reported in similar studies. 16, 17 Use of milk and animal blood were believed to increase the potency of medicinal plants 16 while milk is reported as antidote in case of toxicity and stomach upset. 18

The result of ICF analyses indicated highest value to the disease category that included snakebite, malaria, common cold and related diseases. The use of a number of plants to handle a certain disease and the high agreement among several respondents implies high prevalence of the disease. Largest number of medicinal plant species (13 species) were cited to treat single type of disease (snakebite) while nine species were mentioned to treat more than one disease (malaria, common cold, cough and fever). Hence, snakebite is the prevalent disease identified in the study area, which is similar to findings of studies conducted elsewhere in the region. 16

Cyphostemma adenocaule is identified as the most preferred species used to treat snakebite. This result is supported by the FL analysis. A review report by 41 included the species as one of the medicinal plant species used by local communities of Afar as well as the neighboring Tigray and Oromiya regions. Another species belonging to the same genus was reported as useful herb against snakebite Yalo district of Afar region. 18 The bioactive constituents having detoxifying effect might have been confined at genus level. *Zaleya pentandra* was also reported as useful plant against snakebite, which agree with the report by 16. These findings suggest that such shared knowledge might be the reflection of interaction with the actual biodiversity in the area.

Despite these findings, snakebite has not been reported among the list of health problems by the modern health care system. This evidenced that the problem is controlled by herbal medication system. The contribution of these species could have been extended to tackle other related health problems. Evaluating and validating the efficacy can promote the benefit of the species.
